# Environmental factors and spatiotemporal distribution of Japanese encephalitis after vaccination campaign in Guizhou Province, China (2004–2016)

**DOI:** 10.1186/s12879-021-06857-3

**Published:** 2021-11-22

**Authors:** Suye Zhao, Yidan Li, Shihong Fu, Ming Liu, Fan Li, Chunting Liu, Jing Yu, Liping Rui, Dingming Wang, Huanyu Wang

**Affiliations:** 1 Guizhou Provincial Center for Disease Control and Prevention, 101, Ba Ge Yan road, Yunyan District, Guiyang, 550004 Guizhou China; 2 State Key Laboratory of Remote Sensing Science, College of Global Change and Earth System Science, Beijing Normal University, Beijing, 100875 China; 3 School of National Security and Emergency Management, Beijing Normal University, Beijing, 100875 China; 4 Department of Viral Encephalitis, NHC Key Laboratory of Biosafety, National Institute for Viral Disease Control and Prevention, Chinese Center for Disease Control and Prevention, 155 Changbai Road, Changping District, Beijing, 102206 China; 5 State Key Laboratory of Infectious Disease Prevention and Control, National Institute for Viral Disease Control and Prevention, Chinese Center for Disease Control and Prevention, 155 Changbai Road, Changping District, Beijing, 102206 China

**Keywords:** Japanese encephalitis, Spatial analysis, Spatial epidemiology, Mosquito, Vaccine

## Abstract

**Background:**

Although a vaccination campaign has been conducted since 2004, Japanese encephalitis (JE) is still a public health problem in Guizhou, one of the provinces with the highest incidence of JE in China. The aim of this study was to understand the spatiotemporal distribution of JE and its relationship with environmental factors in Guizhou Province in the post-vaccination era, 2004–2016.

**Methods:**

We collected data on human JE cases in Guizhou Province from 2004 to 2016 from the national infectious disease reporting system. A Poisson regression model was used to analyze the relationship between JE occurrence and environmental factors amongst counties.

**Results:**

Our results showed that the incidence and mortality of JE decreased after the initiation of vaccination. JE cases were mainly concentrated in preschool and school-age children and the number of cases in children over age 15 years was significantly decreased compared with the previous 10 years; the seasonality of JE before and after the use of vaccines was unchanged. JE incidence was positively associated with cultivated land and negatively associated with gross domestic product (GDP) per capita, vegetation coverage, and developed land. In areas with cultivated land coverage < 25%, vegetation coverage > 55%, and urban area coverage > 25%, the JE risk was lower. The highest JE incidence was among mid-level GDP areas and in moderately urbanized areas.

**Conclusions:**

This study assessed the relationship between incidence of JE and environmental factors in Guizhou Province. Our results highlight that the highest risk of JE transmission in the post-vaccination era is in mid-level developed areas.

**Supplementary Information:**

The online version contains supplementary material available at 10.1186/s12879-021-06857-3.

## Background

Japanese encephalitis (JE) is a zoonotic disease caused by the Japanese encephalitis virus (JEV). JE mainly causes damage to the central nervous system, with a disability rate about 30–50% and fatality rate is about 20–30%, which seriously threaten human health [1, 2]. JE is prevalent mainly in Asia and the Western Pacific. About 68,000 cases of JE occur annually, the mortality is high (about 17,000 deaths) [3]. The widespread use of JE vaccines has greatly reduced the incidence of JE in epidemic areas [4, 5]. For example, the reported number of Japanese and Korean cases of JE have dropped to double digits, or even lower [6, 7]. China was once a high epidemic area of JE, and several JE outbreaks occurred in the 1960s and 1970s [8, 9]. After 1998, the JE incidence in China dropped to 1/100,000, with an overall decline across the whole country. However, the regional patterns and age structure of JE onset have also changed [10–12]. JE incidence is rebounding in response to global climate change, leading to increased virus population mobility and changing dominant viral genotypes of epidemic strains, resulting in emerging local outbreaks or epidemics [6, 12–14]. Therefore, JE is still a serious public health problem.

Guizhou Province is an area with one of the highest JE incidences in China [15]. The complex geographical environment in Guizhou is suitable for mosquito breeding. Various arboviruses, including JEV, have been isolated from local mosquitoes [16–18], and JEV and JE IgM antibody have been detected in patient specimens [15]. The epidemic of JE began to be reported in Guizhou Province in 1952. From 1952 to 2003, epidemic reports were reported every year, affecting all counties and cities. For many years, JE vaccine belongs to paid vaccine in our province, so the vaccination rate is low. In order to improve the coverage rate of population immunization, reduce the susceptible population and reduce the incidence of JE, according to the epidemic situation of Guizhou Province and the vaccination situation of JE in past years, Since 2004, live attenuated JE vaccine (SA14-14-2) has been vaccinated in key areas for five consecutive years before the epidemic season of JE. The free vaccination target is children aged from 8 months to 10 years. Since 2006, JE vaccine has been included in the immunization program. Children born after January 1, 2006 can receive free JE vaccine according to the immunization program. This routine vaccination program has continued to this day. During this period, in order to further reduce the incidence of JE in our province, in 2011, on the basis of routine immunization, we launched an intensive immunization campaign against JE vaccine for target children born in key areas from January 1, 2006 to December 31, 2007. Nevertheless, since 1978, the JE incidence in Guizhou Province has remained higher than the national average.

Environmental factors are considered to influence JE transmission via their effect on the main vector of JEV, *Culex tritaeniorhynchus*. Both pig raising and rice farming near human habitats have been found to increase the prevalence of JEV in Asian countries, such as India, Sri Lanka, South Korea, and Nepal [19–22]. In China, relevant studies have shown that climate, altitude, and anthropogenic factors can influence both mosquito abundance and JE transmission [9, 23–25]; however, studies at fine spatial scales are relatively rare. Although vaccination has achieved remarkable results in the control of JE transmission, the disease remains prevalent in Guizhou Province. Therefore, it is essential to evaluate the effects of various factors on disease transmission after implementation of vaccination.

Here, we analyzed the relationship between the disease characteristics and changes in disease distribution among different regions and explored the factors affecting JE transmission. We used spatial–temporal analysis and Poisson regression to detect clustering distributions in space and time at the county (district) level in Guizhou Province, China from 2004 to 2016 and calculated the quantitative influence of dominant environmental factors on JE risk.

## Methods

### Study area

Guizhou Province is located in Southwest China (coordinates range from 24° 37′ N to 29° 13′ N and 103° 36′ E to 109° 35′ E). The province has high elevation variance, with high areas including a mountain plateau in the west, and low areas in the east. A total 92.5% of the area is mountainous and hilly; Guizhou is the only province with no plain areas. Guizhou has a humid subtropical monsoon climate, with a vegetation coverage of 41%, an average sunshine duration of 1100–1600 h, and an average annual accumulated rainfall of 95,402 mm [26, 27]. The total area of the province is 176,100 km^2^, of which minority autonomous areas account for 55.5%. At the end of 2016, the resident population was approximately 35.5 million, of which 19.8 million were rural residents (Guizhou Yearbook). Guizhou Province is divided into nine prefectural administrative regions: Guiyang, Anshun, Bijie, Liupanshui, Qiannan, Qiandongnan, Qianxinan, Tongren, and Zunyi, with 88 counties.

### Data collection

Case report data were derived from the Chinese Center for Disease Control and Prevention’s information system. All data were analyzed anonymously, including the geo-information for each case. We used the reference diagnostic criteria for Japanese encephalitis in China as our definition of JE (WS214-2008) [28]. Cases were clinically diagnosed in patients who lived in JE epidemic areas and whose symptoms occurred during the mosquito breeding season, or in those who traveled to epidemic areas 25 days before disease onset. Symptoms for suspected JE include acute onset, fever, headache, ejection vomiting, and altered consciousness 2–3 days after fever onset. There are different degrees of consciousness disorder; severely affected patients can have generalized convulsions, spasms, or paralysis and other central nervous symptoms, central respiratory failure, absence of superficial reflexes, hyperreflexia, meningeal irritation, pathological reflexes, spastic paralysis, or cerebral ankylosis accompanied by intracranial pressure, alteration of pupil size, elevated blood pressure, decreased heart rate, elevated white blood cell count to 10–20 × 10^9^/L, > 80% neutrophils, increased intracranial pressure, and clear cerebrospinal fluid (CSF) appearance. Laboratory-confirmed JE cases were defined as a suspected JE case for which there was laboratory confirmation by serological testing with JEV-specific IgM in a single CSF or serum sample, or other testing such as virus isolation, reverse transcriptase PCR (RT-PCR), and/or real-time RT-PCR.

Data on annual cumulative, precipitation, and annual average temperature were obtained from the WorldClim database version 2 (http://worldclim.org/version2). This database is a global bioclimatic data map of 1-km resolution. Each grid of map represents the average data for the past 30 years (1970–2000). The overall 18 bioclimatic data were provided. We overlapped the bioclimatic map and county-level administrative map of Guizhou Province and then extracted the data for each county by ArcGIS. Data of GDP per capita were obtained from the Guizhou Province Statistical Yearbook. Land-use data were extracted from the GlobCover 2009 land cover map with a spatial resolution of 300 m, provided by the Université Catholique de Louvain and the European Space Agency. Land use was classified into four types: cropland, vegetation (including trees, herbaceous plants, and shrubs), urban area, and water body. An additional data file shows description of social and environmental condition of Guizhou Province in more detail [see Additional file 1]. Spatial data were analyzed using ArcGIS 10.1 (Esri Inc., Redlands, CA, USA).

### Spatiotemporal analysis

To identify geo-spatial clusters of JE infection, we applied the spatial scan statistic using SaTScan software. All cases were geocoded by residential address at the district or county level. We then calculated the JE incidence rates for each township using national census data from 2004, 2010, and 2015. Spatial and temporal clusters of not more than 30% of the total population at risk were explored and 999 Monte Carlo replicates were used to test the null hypothesis [29]. To assess spatial autocorrelation of JE incidence in Guizhou Province, the global Moran’s I index and Moran Local Indicators of Spatial Association (LISA) were calculated using GeoDa Version 1.12. The weight matrix was generated using Queen contiguity at county level when identifying the spatial clustering [30, 31]. A P-value ≤ 0.05 was considered statistically significant.

### Data analyses

Univariable and multivariable Poisson regression models were fit to detect relationships between environmental indictors and JE risk. The accumulated JE cases from 2004 to 2016 were integrated into district or county levels. Environmental indicators consisted of three parts: climatic factors, land-cover data, and economic conditions. To quantify the extent of factor impacts on JE risk, the incidence rate ratio was defined as the incidence rate variation by a given unit of distinct variables. The number of cumulative cases of each county were used as the dependent variable and population size as offset variable in the Poisson regression. We first conducted the univariate Poisson regression between each variable above and JE incidence. The variables of significant effects (P < 0.05) were selected into multivariable Poisson regression to explore the net effect of each variable on JE risk. We divided all continuous variables into three categories by quartile (i.e. high level (top 25%), middle level (25–75%), low level (last 25%)) and reported the cumulative incidence for each distinct category to assess the results of regression analyses under the assumption of continuousness. These categorized continuous variables were then fit into a Poisson regression. All our analyses were conducted using Stata 9.0 software (StataCorp LP, College Station TX, USA).

## Results

### Epidemiological features of JE cases

**Fig. 1:**
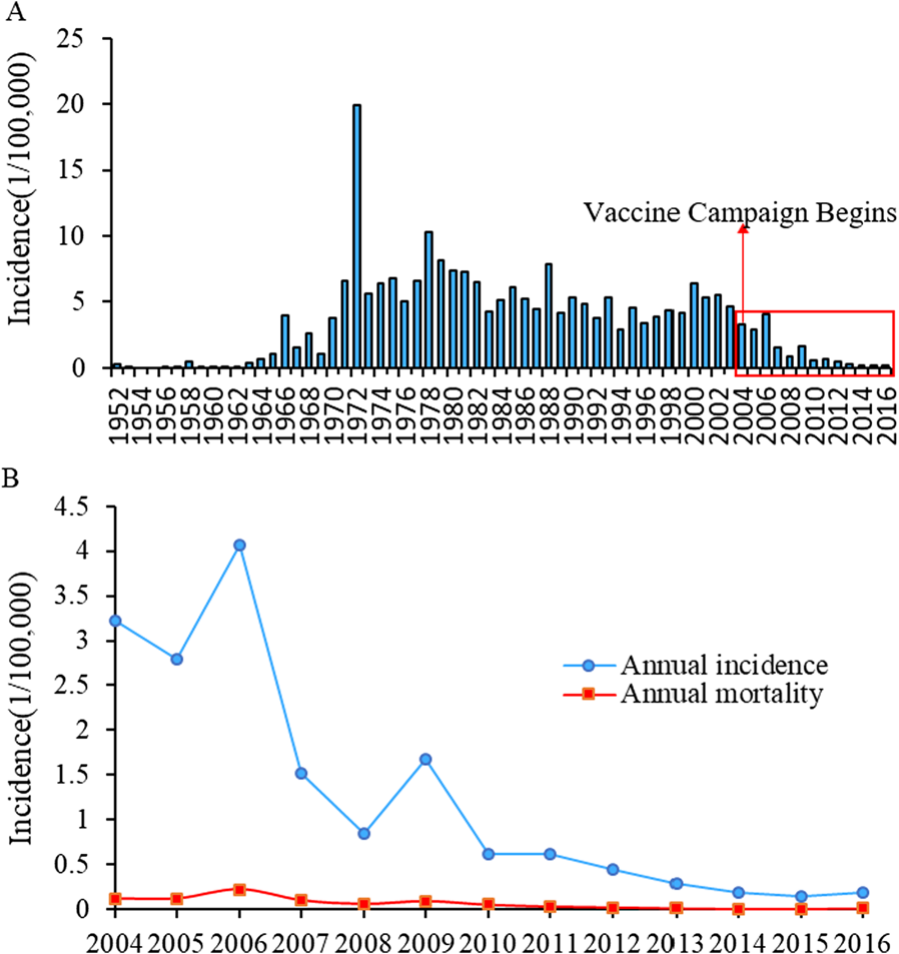
Japanese encephalitis (JE) in Guizhou Province, 2004–2016. **A** Incidence of Japanese encephalitis in Guizhou Province, 1952–2016. Scope of this study marked with red box and time of comprehensive vaccination campaign marked with arrows. **B** Annual morbidity and mortality of JE in 2004–2016. Annual incidence, blue line; annual mortality, red line

There were 7423 JE cases reported in Guizhou Province between 2004 and 2016; the cumulative incidence was 20.96 (1/100,000), with 4664 male and 2759 female patients, reflecting a sex ratio of 1.69:1. Annual incidence rates were between 0.15/100,000 and 4.07/100,000, annual mortality rates between 0.003/100,000 and 0.22/100,000, and the average annual morbidity and mortality were 1.28/100,000 and 0.06/100,000, respectively. From 2004 to 2016, the incidence of JE in Guizhou Province showed an overall downward trend (Fig. 1A, B).

**Fig. 2:**
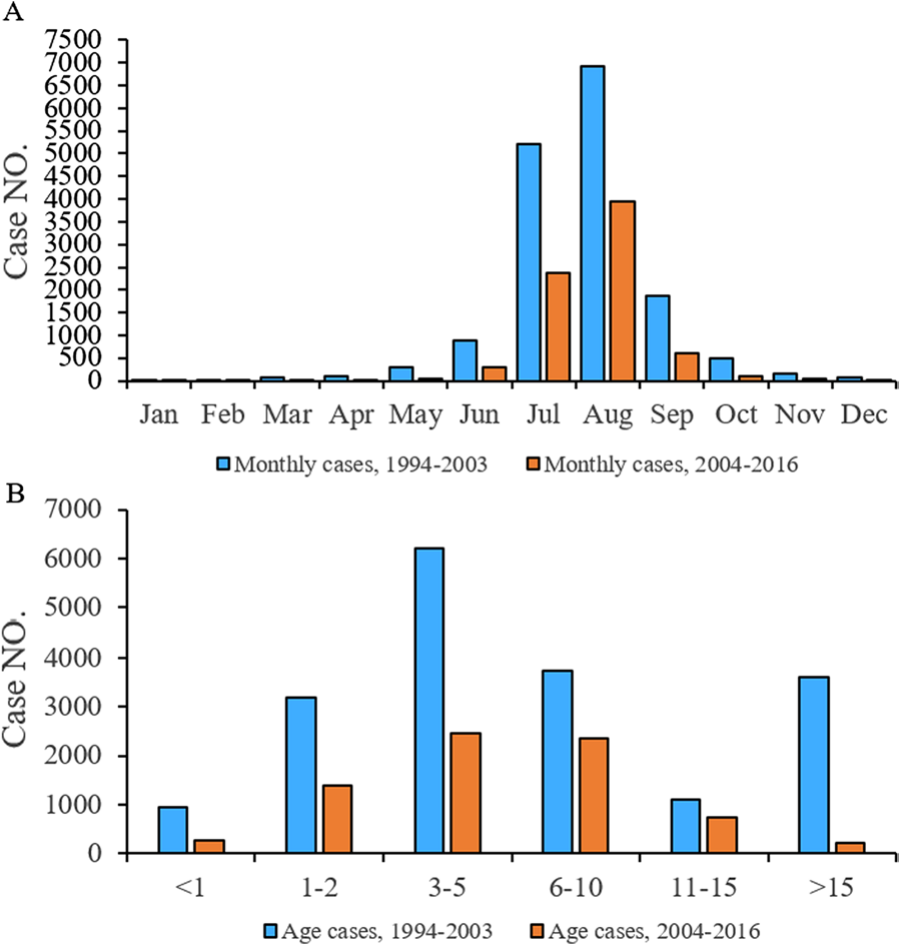
Cumulative distribution of Japanese encephalitis (JE) cases by month and age group, Guizhou Province. Pre-vaccination era (1994–2003) is colored blue and the post-vaccination era (2004–2016) is colored orange. **A** Monthly cumulative distribution, 1994–2003 and 2004–2016. **B** Cumulative distribution by age groups, 1994–2003 and 2004–2016

The incidence of JE exhibited a clearly seasonal distribution, and peaked in July and August. In 1994–2003 and 2004–2016, the cumulative cases in July and August accounted for 75.31% and 85.25% of the total cases, respectively, and the cumulative cases in August reached the peak, accounting for 42.95% and 53.32% of the total cases, respectively (Fig. 2A). In 1994–2003, JE cases were mainly concentrated in the age group 3–5 years, followed by the age groups 6–10 years and > 15 years. In 2004–2016, JE cases were concentrated in children aged 1–10 years, with the most cases in the age group 3–5 years followed by 6–10 years (Fig. 2B).

**Fig. 3:**
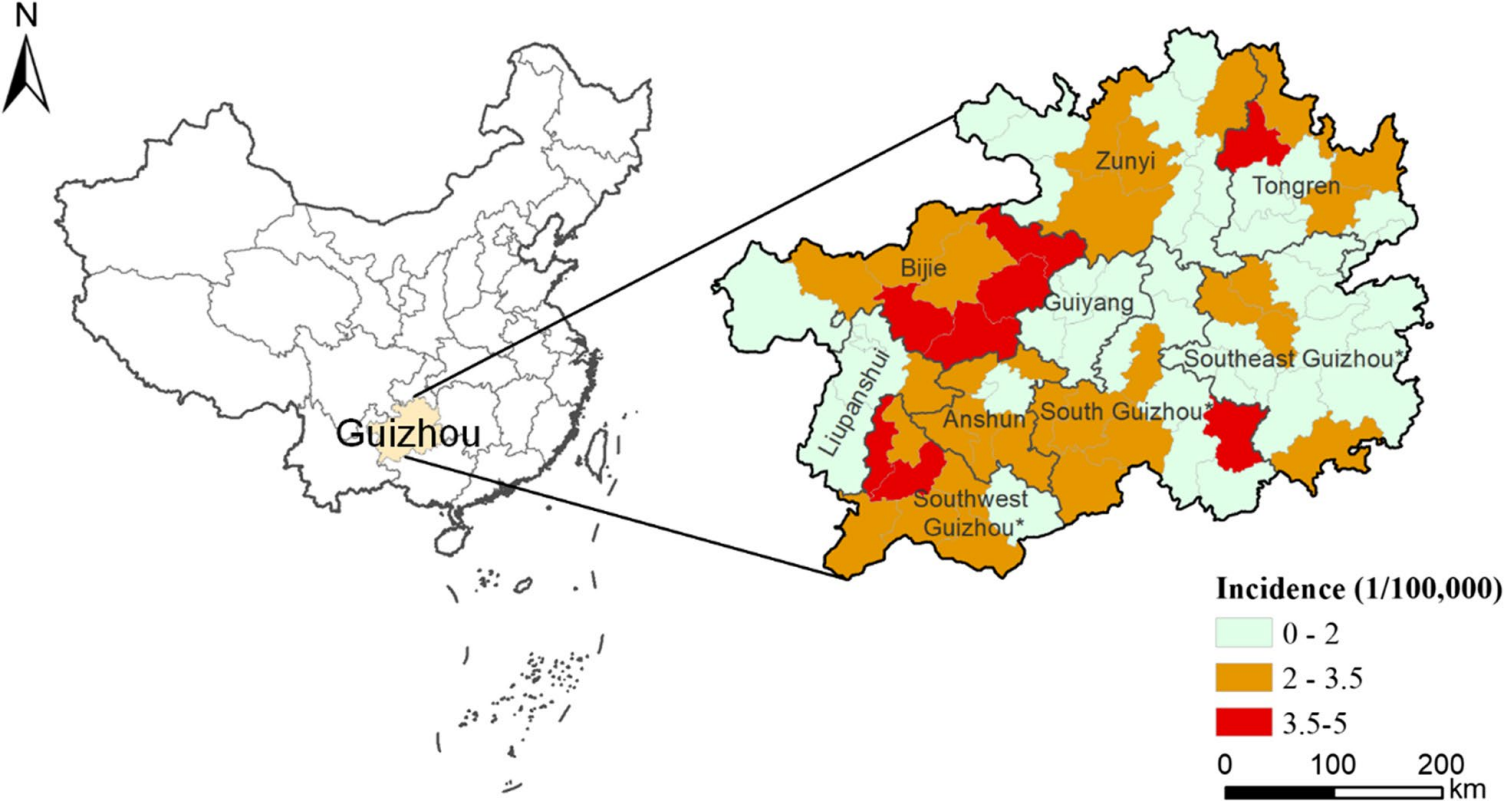
Japanese encephalitis (JE) incidence at county level in Guizhou Province. City names are labeled. *Represents minority autonomous prefecture. Incidence is shaded from low (light green) to high (red). The map was created by the base map provided by ArcGIS system

The JE incidence at county level in Guizhou Province between 2004 and 2016 is shown in Fig. 3. The eight counties with the highest incidences in Guizhou Province were: Dejiang County of Tongren (north); Pu’an County and Xingren County of Qianxinan (west); Nayong County, Zhijin County, Qianxi County, and Jinsha County of Bijie (northwest); and Sandu County of Qiannan (south) (Fig. 3).

### Spatiotemporal distribution of JE cases

**Fig. 4:**
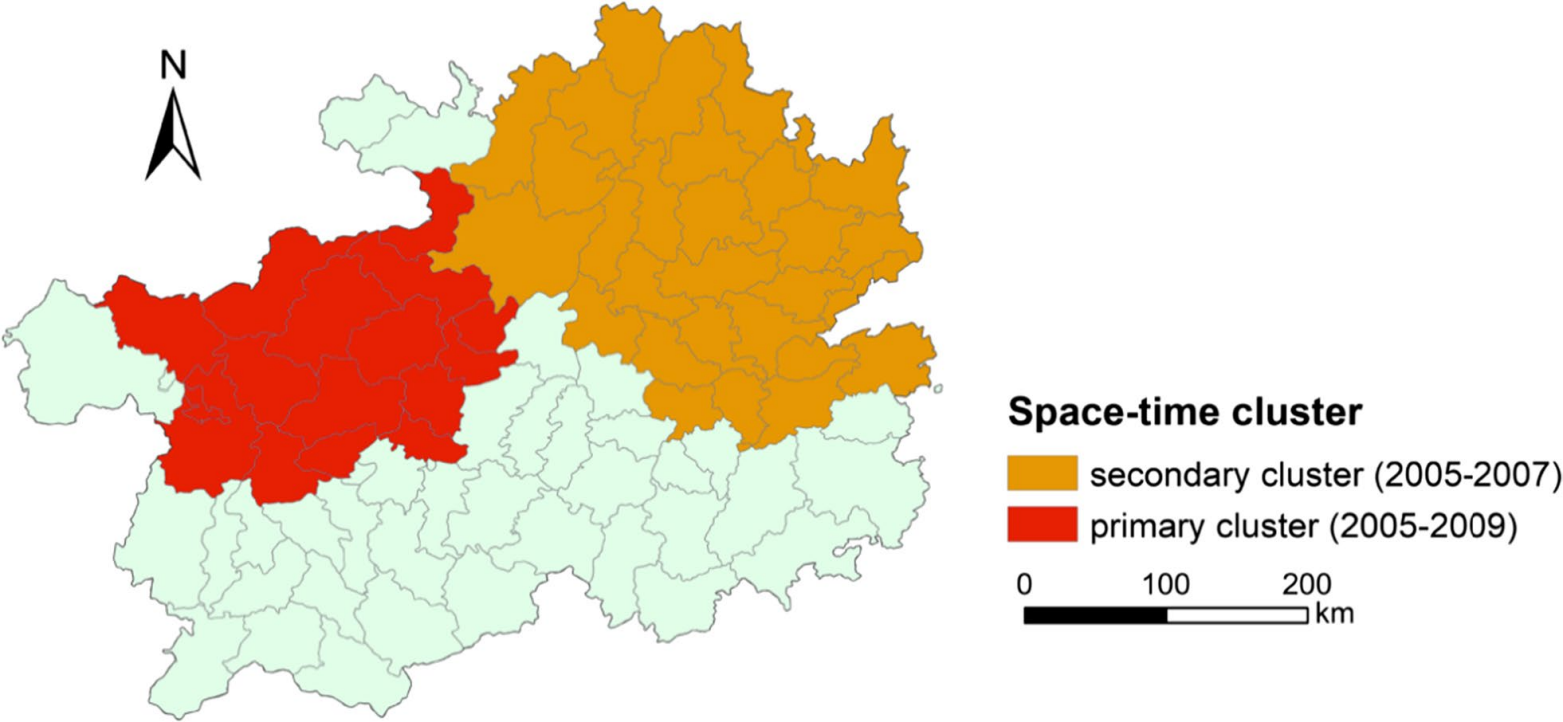
Space–time clusters with high incidence of Japanese encephalitis (JE) in Guizhou Province, 2004–2016. The area of the primary cluster (2005–2009) is indicated in red. The area of the secondary cluster (2005–2007) is indicated in orange. The map was created by the base map provided by ArcGIS system

Two space–time clusters of high JE incidence in the west and east of the province were detected separately using SaTScan (Fig. 4). The 16 county-level regions in northwestern Guizhou Province were found to be the primary cluster of JE risk, with time range from 2005 to 2009. This cluster was concentrated in Dafang County, mainly including Bijie, the northwestern region of Guiyang, and the northern region in Liupanshui. The secondary cluster detected was located in northeastern Guizhou Province during 2005–2007, including 29 county-level regions. Table 1 provides the detailed results of spatial scan analysis.

**Table 1:** Space–time cluster analysis of Japanese encephalitis in Guizhou Province

Cluster	Cluster year	Cluster Center			Radius (km)	Num	LLR	RR
		County	Lat	Lon				
Primary cluster	2005–2009	Dafang County	27.23	105.69	120.86	17	1013.14	3.49
Secondary cluster	2005–2007	Yinjiang Tujia and Miao Autonomous counties	28.00	108.52	167.69	29	316.95	2.39

*Lat.* latitude; *Lon.* longitude; *Num.* number of clustered counties; *LLR* log-likelihood ratio; *RR* relative risk

P < 0.001

**Fig. 5:**
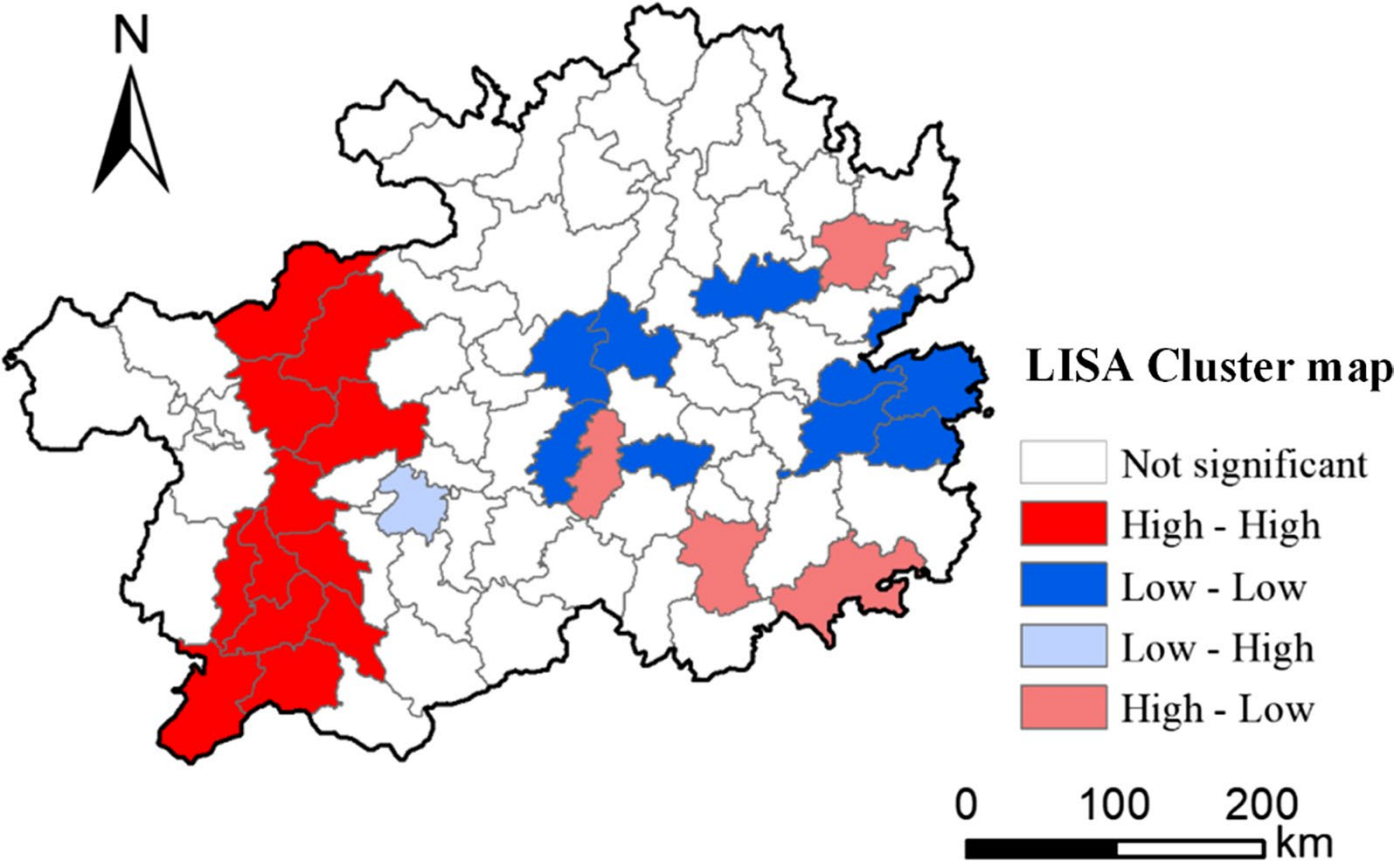
Moran LISA cluster map of Japanese encephalitis (JE) cumulative incidence in Guizhou Province, 2004–2016. Colored regions indicate the spatial clusters of significance (P < 0.05). Four categories of hotspots are indicated: High-High in red, Low-Low in blue, Low–High in pale blue, High-Low in pink. LISA, Local Indicators of Spatial Association. The map was created by the base map provided by ArcGIS system

From 2004 to 2016, the incidence of JE had spatial autocorrelation with significant clustering characteristics (global Moran’s I = 0.35, P < 0.05). LISA indicated the presence of significant spatial clusters (Fig. 5): (1) high-incidence clusters in the west and northwest (Bijie municipal district, Dafang County, Nayong County, Zhijin County, Liuzhi County, Guanling County, Qinglong County, Pu’an County, Xingren County, Zhenfeng County, Anlong County, and Xingyi City); (2) low-incidence clusters in the central province and the east (Kaiyang, Weng’an, Longli, Majiang, Yuping, Sansui, Jianhe, Tianzhu, and Jinping); (3) high-incidence areas (Guiding, Sandu, Congjiang, and Jiangkou) surrounded by low-incidence areas; and (4) low-incidence areas surrounded by high-incidence areas (Anshun municipal district).

### Environmental factors in JE incidence

The Poisson regression model (Table 2) indicated that JE incidence was significantly associated with GDP per capita, cropland coverage, vegetation, and built-up land, but not average temperature and precipitation. The results suggest that the difference in JE incidence across counties in Guizhou Province between 2004 and 2016 may not be induced by climatic factors. In particular, JE incidence was negatively associated with GDP per capita; the middle economic class had the highest risk. The incidence rose with increasing cropland but dropped with increasing coverage of built-up land. According to our results, cropland coverage < 25%, vegetation coverage > 55%, and urban-area coverage > 25% were the lowest-risk landscapes for JE. Specifically, the JE incidence in relatively high-coverage croplands (> 35%) was 1.58 times that of low-coverage croplands (< 25%). The JE incidence of high-use urban lands (> 0.25%) was 0.8 times that of low-use urban lands (< 0.25%).

**Table 2:** Relationship between environment factors and Japanese encephalitis incidence in Guizhou Province, 2004–2016

Variables (unit)	Cumulative incidence (95% CI, per 100,000 persons)	IRR (95% CI)	IRR (95% CI)
		Univariate analysis	Multivariate analysis
GDP per capita (categorical, 1000 Yuan)			
< 7	21.06 (16.44, 25.69)	–	–
7–12	23.45 (19.96, 26.93)	–	–
> 12	17.37 (12.84, 21.89)	–	–
GDP per capita (continuous, 1000 Yuan)	–	0.99 (0.98, 0.99)**	0.97 (0.96, 0.98)**
Precipitation (categorical, 10 mm)			
< 110	24.31 (17.02, 31.58)	–	–
110–125	19.88 (17.24, 22.52)	–	–
> 125	23.82 (16.04, 31.60)	–	–
Precipitation (continuous, 10 mm)	–	1 (0.99, 1.01)	–
Average temperature (categorical, 1 °C)			
< 14	24.12 (16.57, 31.68)	–	–
14–15.8	20.42 (17.08, 23.76)	–	–
> 15.8	21.56 (17.56, 25.57)	–	–
Average temperature (continuous, 1 °C)	–	1 (0.97, 1.04)	-
Croplands (categorical, 1%)			
< 25	19.96 (17.15, 22.78)	1	1
25–35	21.11 (15.72, 26.50)	1.06 (0.94, 1.19)	0.89 (0.78, 1.03)
> 35	30.84 (22.69, 38.98)	1.54 (1.35, 1.77)**	1.58 (1.47, 1.68)**
Vegetation (categorical, 1%)			
< 30	24.77 (19.77, 29.79)	1	1
30–55	21.89 (18.51, 25.26)	0.88 (0.79, 0.98)*	0.79 (0.69, 0.92)**
> 55	15.80 (11.54, 20.06)	0.64 (0.55, 0.73)**	0.57 (0.47, 0.68)**
Urban areas (categorical, 1%)			
< 0.12	21.64 (17.13, 26.15)	1	1
0.12–0.25	23.36 (19.01, 27.70)	1.08 (0.95, 1.21)	1.08 (0.95, 1.22)
> 0.25	19.04 (15.15, 22.92)	0.87 (0.78, 0.99)*	0.80 (0.68, 0.94)*
Water bodies (categorical, 10%)			
< 0.08	17.81 (12.70, 22.92)	1	1
0.08–2.1	20.95 (16.67, 25.22)	1.22 (1.08, 1.38)**	1.10 (0.97, 1.26)
> 2.1	23.59 (19.96, 27.23)	1.20 (1.04, 1.37)*	1.02 (0.87, 1.21)

*CI* confidence interval; *IRR* incidence rate ratio; *GDP* Gross Domestic Product

*P < 0.05; **P < 0.01

## Discussion

JE is a common mosquito borne disease in the tropics and subtropics, where the growth and reproduction of mosquitoes are enhanced by local environmental factors [13, 32–34]. Herein, using GIS technology and regression analysis, we aimed to identify aggregated spatial or temporal distributions of JE cases in Guizhou Province, and to explore whether these aggregations are associated with environmental factors.

During 1994 and 2016, JE cases increased significantly in July and reached the peak in August. This seasonal characteristic did not change with vaccination campaigns. But the increased incidence of JE in July and August may also be related to summer vacation in school. The age group 3–5 years had the highest incidence of JE, indicating that young children may play in the fields in closer proximity with the animal reservoirs, they are more likely to be bitten by mosquitoes and infected with JE; they are younger and therefore less likely to be immunologically experienced to previous asymptomatic JE infections and therefore more susceptible to symptomatic disease after first infection. The age group 6–10 years had the second highest incidence, indicating that school children are likely to be outdoors and playing during the peak mosquito population period, thus exposing themselves to JE infection suggesting that supplementary immunization activities should be considered for school-age children. In addition, the number of cases in the age group over 15 years in the period 2004–2016 was significantly decreased compared with the number during 1994–2003, which may be a result of immunization activities.

Vector-borne diseases are known to be affected by climatic factors, such as temperature and precipitation [35–37]. It has been reported that temperatures about 22 °C and relative humidity 70–74% favor JE transmission [38, 39]. The average temperature in the epidemic period of JE in Guizhou Province is about 22–25 °C, and the relative humidity is over 70%. The variation in temperature and relative humidity among counties of the province is relatively small, which may be the reason that temperature and precipitation were not statistically significant in the model.

GDP per capita is an important indicator for measuring the economic status of a region [40], and our results showed that JE incidence is negatively associated with GDP. JE incidence was lower in areas with high GDP per capita, which is consistent with previous reports [41]. In the past, the economic development of Guizhou Province was relatively poor, especially in the northwest, and the JE incidence was relatively high. With increased economic development, JE incidence has been gradually decreasing. This trend may be owing to the gradual growth in GDP, the corresponding increase in national investment in infrastructure and public health, and improvement in residents’ quality of life and health awareness, especially the increase in disease-prevention spending by non-wealthy residents. However, it should be noted that JE incidence is highest at the mid-level GDP per capita. We suspected that residents in low-level GDP per capita areas may travel to areas with mid-level GDP per capita that have better medical services and more moderate medical costs, consequently driving up the risk of JE in mid-level GDP per capita regions. Therefore, we suggest that greater attention is needed for disease prevention and control in areas with mid-level GDP per capita.

It has been reported that the risk of JE is related to paddy fields and pig farming [22, 42, 43]. Guizhou is a major agricultural region. Crop production is the primary industry, and rice planting is common, but the distribution of agricultural landscapes varies across regions. The northwest part of the province has high elevation and dryland, drought-resistant crops such as corn and potatoes are widely cultivated there. There are many paddy fields in the south and east, where rice is widely cultivated. Agricultural land area was found to be positively correlated with JE occurrence, which could be because local farmers usually live near farmland and raise pigs and cattle near their home [44]. For example, the Buyi and Miao people, who set up dwellings near the mountains and water bodies, build two-layer tower structures in which household members live in the upper levels and the lower level serves as a fence for livestock, farm equipment storage, and household debris. Some other minority groups mix human and livestock waste, which probably increases the frequency of mosquito bites, and thus, JE exposure. High vegetation coverage is generally thought to increase JE incidence; however, our results suggest a negative relationship between vegetation area and JE incidence. We speculate that the smaller population residing in mountainous areas with high vegetation coverage may reduce the opportunity for mosquito–human contact, and thus, lower the JE reporting and observation rates in Guizhou Province.

Human activities have an impact on the lifecycle of mosquito. Our results showed a negative correlation between urban land area and JE incidence. Agricultural land area accounts for about 85% of the total land area in Guizhou [38]. There are many ethnic minorities living in the countryside, and some of their unique traditional behaviors may increase the risk of disease. With urban and rural integration in Guizhou Province, the size of urban areas has been expanding, which has reduced the range of mosquitos and the risk of natural infection with JEV. Today, rural residents’ living areas are more frequently separated from their livestock, and with implementation of a series of policies ensuring better sanitation and land restoration, successful prevention and control of JE in Guizhou Province has been partly achieved. Nevertheless, greater attention must be focused on mid-level urbanized areas, which have the highest JE incidence. These areas are usually in various stages of rapid development and population mobility. Environmental changes are great and may be accompanied by changes in mosquito migration and reproduction. We suggest that strengthening the prevention and control of JE is needed in Guizhou, particularly in mid-level urbanized areas. Such well-planned urbanization can improve people's living environment, increase health awareness, and improve the quality of planned immunization, effectively reducing the JE risk.

## Limitations of the study

This study has several limitations. First, land use in Guizhou Province has undergone several changes during the 13-year study period, so the available land-use data should be updated. Second, climatic data by county was estimated using the WorldClim database, which was created via a spatial interpolation method. Finally, due to the specific-county immunization data is not available, we haven’t examined the effects of vaccine coverage on JEV incidence rate across counties.

## Conclusion

In conclusion, economic level, land use, and urbanization are significantly related to JE risk. These findings may be helpful to identify areas requiring strengthened prevention and control programs. Our work provides an important reference for continued efforts at reducing JE incidence in Guizhou Province.

## Supplementary Information

**Additional file 1: Figure S1.** GDP per capita of counties in Guizhou Province. **Figure S2.** The climate variability including cumulative precipitation, average temperature. **Figure S3.** The land use of urban area, cropland, water body and vegetation coverage of counties in Guizhou Province.

## Data Availability

The datasets analysed during the current study are available from the corresponding author on reasonable request.

## Abbreviations

JE: Japanese encephalitis
JEV: Japanese encephalitis virus
GDP: Gross domestic product
