# Supplementary material for: Environmental factors and spatiotemporal distribution of Japanese encephalitis after vaccination campaign in Guizhou Province, China (2004–2016)
Source: BMC Infect Dis. 2021 Nov 22;21:1172. doi: 10.1186/s12879-021-06857-3 (PMC8607706; doi:10.1186/s12879-021-06857-3)
Supplement: Supplementary file 1 — Additional file 1: Figure S1. GDP per capita of counties in Guizhou Province. Figure S2. The climate variability including cumulative precipitation, average temperature. Figure S3. The land use of urban area, cropland, water body and vegetation coverage of counties in Guizhou Province. [file 12879_2021_6857_MOESM1_ESM.pdf]

Basic environmental and social conditions of Guizhou Province

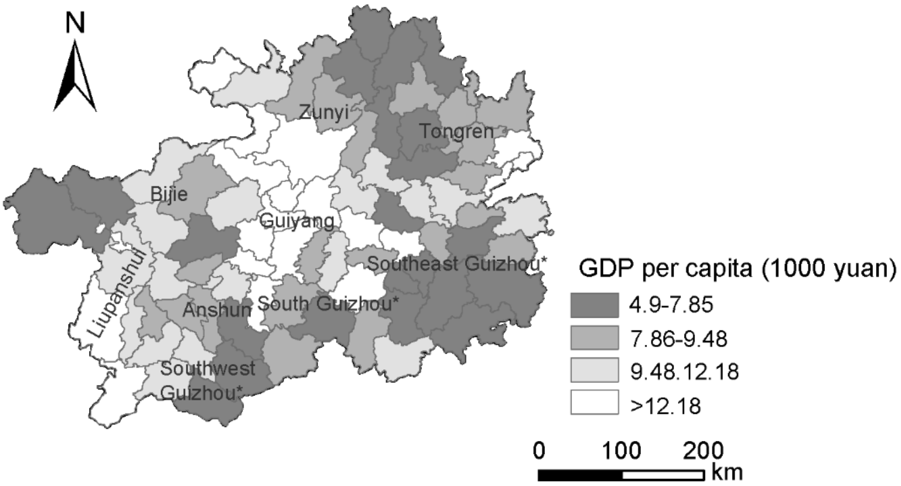

Fig S1. GDP per capita of counties in Guizhou Province. The highest level of GDP in Guizhou Province is the neighboring counties with Guiyang Municipal District, Zunyi Municipal District, Duyun City, Xingyi City and Tongren Municipal District as the center radiation, accounting for 21.6%, the cities with medium level of GDP accounting for 46.6%, and the cities with the lowest level of GDP accounting for 31.8%. The classification method is the same as the method in main text. The map was created by the base map provided by ArcGIS system.

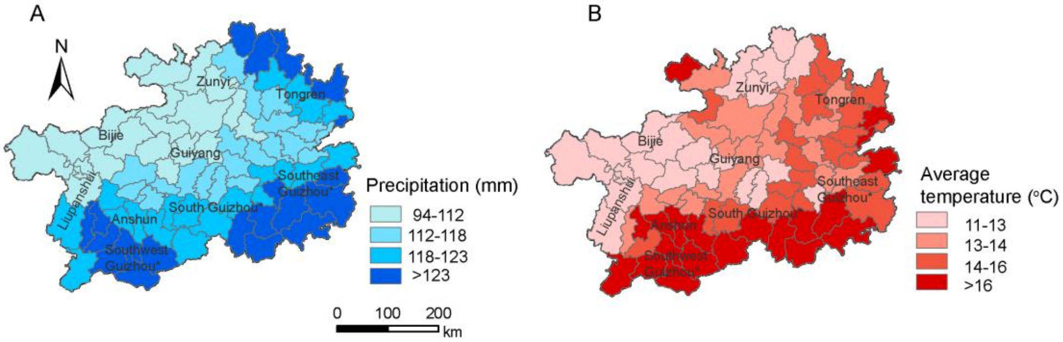

Fig S2. The climate variability including cumulative precipitation, average temperature. The highest cumulative precipitation is in Tongren, Qiannan, Qianxinan and Qiandongnan, and the lowest is in most districts and counties under the jurisdiction of Bijie, Zunyi and Guiyang. The lower average temperature is mainly in Bijie, Liupanshui and parts of Zunyi, and the higher temperature is mainly in Qianxinan, Qiannan, Qiandongnan and Tongren of Guizhou. The map was created by the base map provided by ArcGIS system.

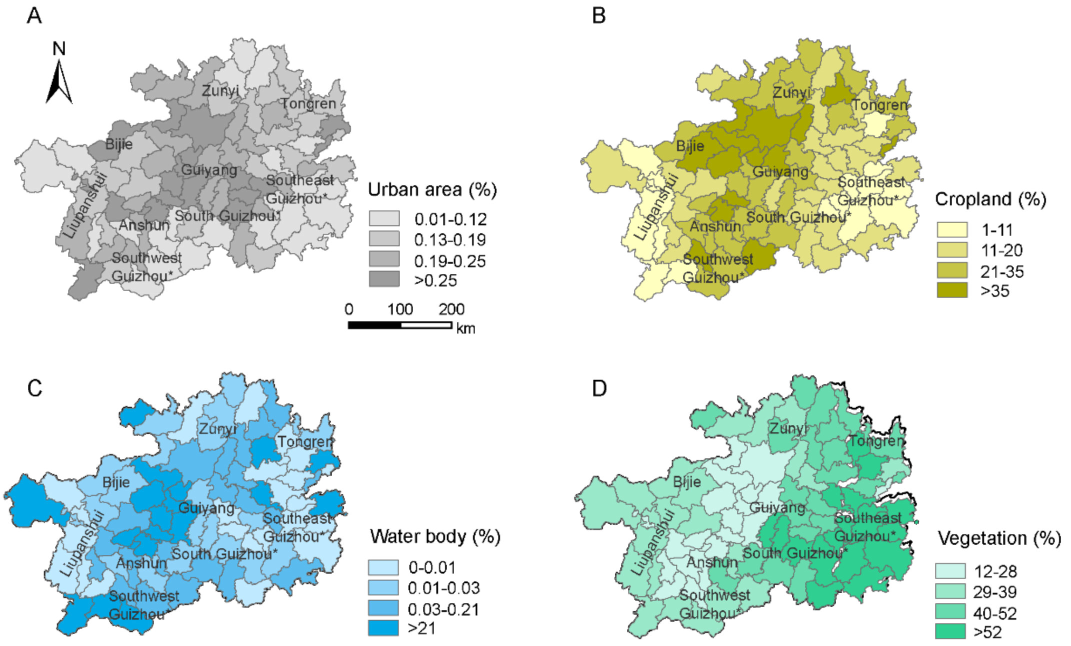

Fig S3. The land use of urban area, cropland, water body and vegetation coverage of counties in Guizhou Province. In Guizhou Province, medium-sized urbanization accounts for more than half of the total, and the highest level of urbanization is in the municipal districts under the jurisdiction of nine cities (prefectures). There are more farmland in the northwest, South and southwest, while the vegetation in the north, South and southeast is the most abundant. The Yangtze River and the Pearl River system diverge from the West and the middle to the north, East and south, and the large and medium-sized water bodies in the province are widely distributed. The map was created by the base map provided by ArcGIS system.
